# Pterostilbene Interferes With Lipopolysaccharide-Induced Myocardial Injury Through Oxidative Stress and Inflammasome Pathways

**DOI:** 10.3389/fphys.2022.862187

**Published:** 2022-03-24

**Authors:** Lei Zhang, Xiao Jian, Jiachuan Yu, Jian Yu

**Affiliations:** ^1^Department of Critical Care Medicine, The Second Affiliated Hospital of Dalian Medical University, Dalian, China; ^2^Department of Nutrition, The Second Affiliated Hospital of Dalian Medical University, Dalian, China; ^3^Department of Anesthesiology, The First Affiliated Hospital of Dalian Medical University, Dalian, China

**Keywords:** pterostilbene, LPS, myocardial dysfunction, reactive oxygen species, inflammasome

## Abstract

Myocardial contractile dysfunction caused by sepsis is a serious threat to human health, and its pathogenesis is not completely clear. It is generally believed that excessive inflammation and oxidative stress are the main causes of myocardial damage caused by sepsis. Pterostilbene (PTS) has a variety of biological activities, including anti-oxidant, anti-inflammatory, and anti-aging. Whether PTS protect myocardial function in rats with sepsis through anti-inflammatory and anti-oxidant effects has not been reported. In this study, we investigated the role of PTS in septic mice induced by lipopolysaccharide (LPS). Mice were injected intraperitoneally with LPS (20 mg/kg) to simulate sepsis. Use Echocardiography, Masson, DHE, H&E, IHC, IF and other experimental methods to explore the effects of PTS on LPS. The results showed that PTS was indicated to significantly increase the cardiac function of mice with sepsis. PTS treatment also reduced the mRNA expression of IL-1α, IL-6, MCP-1, and IL-1β and the protein expression of NLRP3 *in vivo* and *in vitro*, and inhibited the migration of inflammatory cells. PTS treatment also reduced the mRNA expression of collagen I, collagen III and α-SMA, and inhibited fibrosis. PTS treatment reduced the mRNA expression of NOX1, NOX2, and NOX4 and inhibited DHE levels *in vivo* and *in vitro*. In summary, our data indicated that PTS played a crucial role in LPS-induced myocardial injured and might be a key target for the prevention and treatment of sepsis-induced myocardial dysfunction.

## Introduction

Sepsis is the leading cause of death for hospitalized critically ill patients. Death caused by severe sepsis seriously threatens human health and brings a huge economic burden to the public health system. Improving the success of sepsis treatment cannot be delayed. Multiple organ dysfunction is the main cause of death in patients with sepsis, and the heart is one of the most vulnerable targets (Hunter and Doddi, 2010; Poveda-Jaramillo, 2021).

The increase of inflammatory factors, oxygen free radicals and cytotoxic substances are the main cause of myocardial cell energy metabolism disorder and myocardial cell apoptosis (Liu et al., 2015; Andonegui et al., 2018). In sepsis, the inflammatory response always dominates. Bacterial lipopolysaccharide (LPS) activates the intracellular signal transduction mechanism, synthesizes and releases a large number of inflammatory factors, such as tumor necrosis factor-α (TNF-α), interleukin-6 (IL-6), IL-8 and IL-10, and thus causes myocardial injury (Baradaran et al., 2019; Yu et al., 2019). In sepsis, the nicotinamide adenine dinucleotide phosphate (NADPH) oxidase in macrophages and neutrophils could promote the production of reactive oxygen species in the cells, causing damage to myocardial cells and causing a decrease in myocardial contractility. Studies have found that cardiomyocyte apoptosis has occurred in the early stage of sepsis. With the prolongation of the course of sepsis, the number of cardiomyocyte apoptosis gradually increases. It could cause a significant decrease in cardiac systolic function, and even heart failure (Wang et al., 2019; Han et al., 2020).

Pterostilbene (PTS) is an anti-toxic substance similar in structure to resveratrol, mainly found in plants such as blueberries, grapes, and peanuts (Wang and Sang, 2018). In recent years, studies have shown that PTS has a variety of biological activities, including anti-inflammatory, anti-oxidant, anti-aging and anti-viral activities. In addition, PTS could also mediate cell cycle, apoptosis and proliferation (Wang and Sang, 2018). However, the role of PTS in sepsis (Yang et al., 2020), especially in sepsis-induced cardiac dysfunction, is rarely reported. This study intends to explore the role of PTS in sepsis-induced myocardial injury in mice, and to explore its possible mechanism.

## Materials and Methods

### Animals and Treatment

Mice (C57BL/6, ∼24 g, 8–10 w) had free access to water and food. Mice were randomly divided into three groups (Control, LPS, LPS + PTS). A sepsis mouse model was established by intraperitoneal injection of LPS (20 mg/kg). Control group injected sterile saline. At first, there were 10 mice in each group. LPS and LPS + PTS group mice were intraperitoneally injected with PTS (10 mg/kg). After 24 h, LPS was injected intraperitoneally. Then PTS was injected after 2 h. After 24 h, mice were sacrificed. All the procedures involved in the mouse study were approved by the Institutional Animal Care and Use Committee of Dalian Medical University.

### Echocardiography

The cardiac function of mice was measured by ultrasound system after anesthetization with inhaled isoflurane. Ejection fraction (EF%) and fractional shortening (FS%) were calculated and analyzed.

### Hematoxylin-Eosin Staining and Masson

#### Dewaxed and Hydration

Then, use the H&E or Masson kit for staining. After the glass slide is dehydrated with gradient alcohol and xylene, neutral resin is added dropwise to seal the cover glass. After the slides are dry, we observe under a microscope, take pictures.

### Immunohistochemistry

#### Dewaxed and Hydration

The heart tissue sections were incubated with 3% H_2_O_2_ for 10 min, and then washed with PBS three times, tissue blocking for 1 h, incubate with primary antibodies overnight at 4°C, incubate with the secondary antibody, followed by the use of DAB to visualize staining. After the slides are dry, we observe under a microscope, take pictures.

### Real-Time Polymerase Chain Reaction

Extract total RNA with TRIzol reagent. After quantification, the RNA was reverse transcribed into cDNA using a transcription kit polymerase chain reaction (PCR) amplification. The 2^–ΔΔCt^ method was used to calculate the relative expression of the target gene. The primer sequence is shown in Table 1.

**TABLE 1:** Primers used for quantitative real-time PCR analysis.

Gene	Forward primer (5′-3′)	Reverse primer (5′-3′)
IL-1α	TCTATGATGCAAGCTAT GGCTCA	CGGCTCTCCTTGAAGGTGA
IL-1β	TGCCACCTTTTGACA GTGATG	TGATGTGCTGCTGCGAGATT
IL-6	TGATGGATGCTACCAAA CTGGA	TGTGACTCCAGCTTATCTCTTGG
MCP-1	TAAAAACCTGGATCGGAA CCAAA	GCATTAGCTTCAGATTTACGGGT
Collagen I	GAGTACTGGATCGACCCT AACCA	GACGGCTGAGTAGGGAACACA
Collagen III	TCCCCTGGAATCTGT GAATC	TGAGTCGAATTGGGGAGAAT
α-SMA	TCCTGACGCTGAAGTATC CGATA	GGCCACACGAAGCTCGTTAT
NOX1	TTGTTTGGTTAGGGCTGA ATGT	GCCAATGTTGACCCAAGGATTTT
NOX2	ACCGGGTTTATGATATTC CACCT	GATTTCGACAGACTGGCAAGA
NOX4	CAGATGTTGGGGCTAG GATTG	GAGTGTTCGGCACATGGGTA
GAPDH	GGTTGTCTCCTGCGA CTTCA	GGTGGTCCAGGGTTTCTTACTC

*IL-1α, interleukin 1 alpha; IL-1β, interleukin 1 beta; IL-6, interleukin 6; MCP-1, Monocyte Chemotactic Protein 1; α-SMA, α-smooth muscle actin; NOX1, NADPH oxidase 1; NOX2, NADPH oxidase 2; NOX4, NADPH oxidase 4; GAPDH, glyceraldehyde 3-phosphate dehydrogenase.*

### Western Blot

Extract the total protein with RIPA protein lysate Keygenbio, KGP250 on ice and quantify with bicinchoninic acid (BCA). The proteins were separated by sodium dodecyl sulfate polyacrylamide gel electrophoresis (SDS-PAGE), and then transferred to polyvinylidene fluoride (PVDF) membrane. Then, the PVDF membrane was blocked with 5% skim milk at room temperature for 1 h, and the primary antibody NOX2, NOX4, ASC, NLRP3, IL-1β, IL-18, β-actin (Arigo) was incubated overnight at 4°C. The next day, after washing the PVDF membrane with Tris-Buffered Saline and Tween 20 (TBST), add the corresponding secondary antibody. After incubating for 1 h at room temperature, after washing the PVDF membrane with TBST. ECL was added dropwise to image in the gel imaging system.

### Dihydroethidium

#### Dewaxed and Hydration

Incubate the sections with DHE solution in the dark at room temperature for 10 min. After washing, observe under the microscope, take pictures and make statistics.

### Cell Culture

H9C2 cells were cultured in DMEM medium containing 10% Foetal Bovine Serum (FBS, Life-iLab, China) (100 U/ml penicillin and 100 μg/ml streptomycin) and cultured in a 37°C 5% CO_2_ saturated humidity incubator.

### Immunofluorescence

The cells were fixed in paraformaldehyde for 15 min, washing three times with phosphate buffered solution (PBS), for 10 min at room temperature with 0.3% Triton X-100, washing three times with PBS, the cells with 5% goat serum for 30 min at room temperature, and incubate the primary antibody at 4°C overnight. After washing three times, the cells were incubated with the secondary antibody for 1 h at room temperature, washed three times and reacted with 2-(4-Amidinophenyl)-6-indolecarbamidine dihydrochloride (DAPI). Observe under the microscope, take pictures and make statistics.

### Statistical

SPSS 20.0 software was used for statistical analysis. The experimental data were expressed as mean ± standard deviation (Mean ± SD). Comparisons between multiple groups were performed using one-way ANOVA. *P* < 0.05 was considered statistically significant. *P < 0.05, **P < 0.01, ***P < 0.001 control vs. LPS; ^#^*P* < 0.05, ^##^*P* < 0.01, ^###^*P* < 0.001 LPS vs. LPS + PTS.

## Results

### Pterostilbene Improved the Survival Rate and Myocardial Dysfunction in Lipopolysaccharide-Induced Septic Mice

**FIGURE 1:**
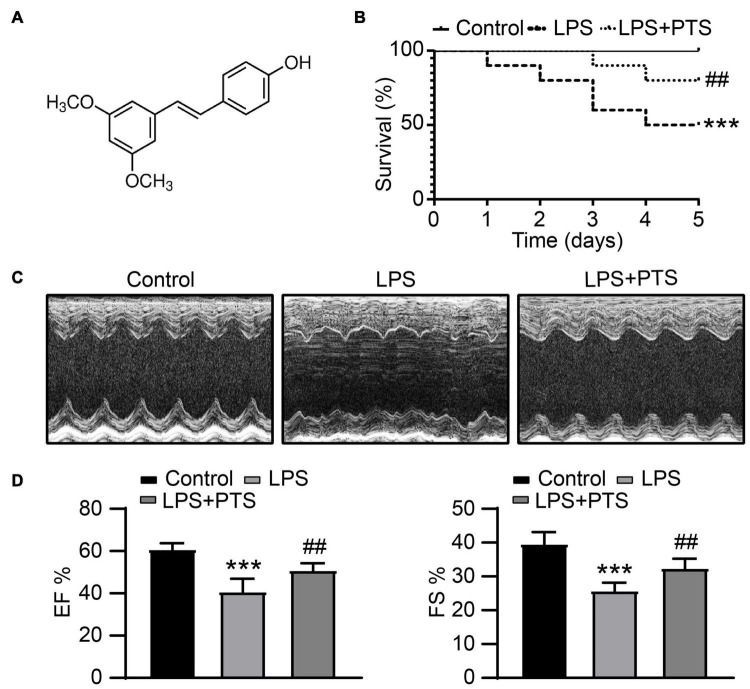
PTS improved the survival rate and myocardial dysfunction in LPS-Induced septic mice. **(A)** The Structural formula of PTS. **(B)** Survival rate of the control group, LPS group, and LPS + PTS group (*n* = 10). **(C)** Representative echocardiography of mice from control group, LPS group, and LPS + PTS group. **(D)** EF and FS% of mice from control group, LPS group, and LPS + PTS group were measured by echocardiography (*n* = 6). ***P < 0.001 control vs. LPS, ^##^*P* < 0.01 LPS vs. LPS + PTS.

The survival rate treated was observed. The result showed that the 5 days survival rate of LPS injection mice was ∼fifty percent, and the survival rate of LPS + PTS mice was ∼eighty percent (Figure 1A). The addition of PTS significantly inhibited mortality in LPS-induced mice (Figure 1B). We used echocardiography to detect the myocardial dysfunction. The results showed that EF and FS% was significantly decreased by LPS, and PTS could improve EF and FS% in LPS-induced mice (Figures 1C,D).

### Pterostilbene Inhibits Inflammation in the Hearts of Mice With Lipopolysaccharide-Induced Septic Mice

**FIGURE 2:**
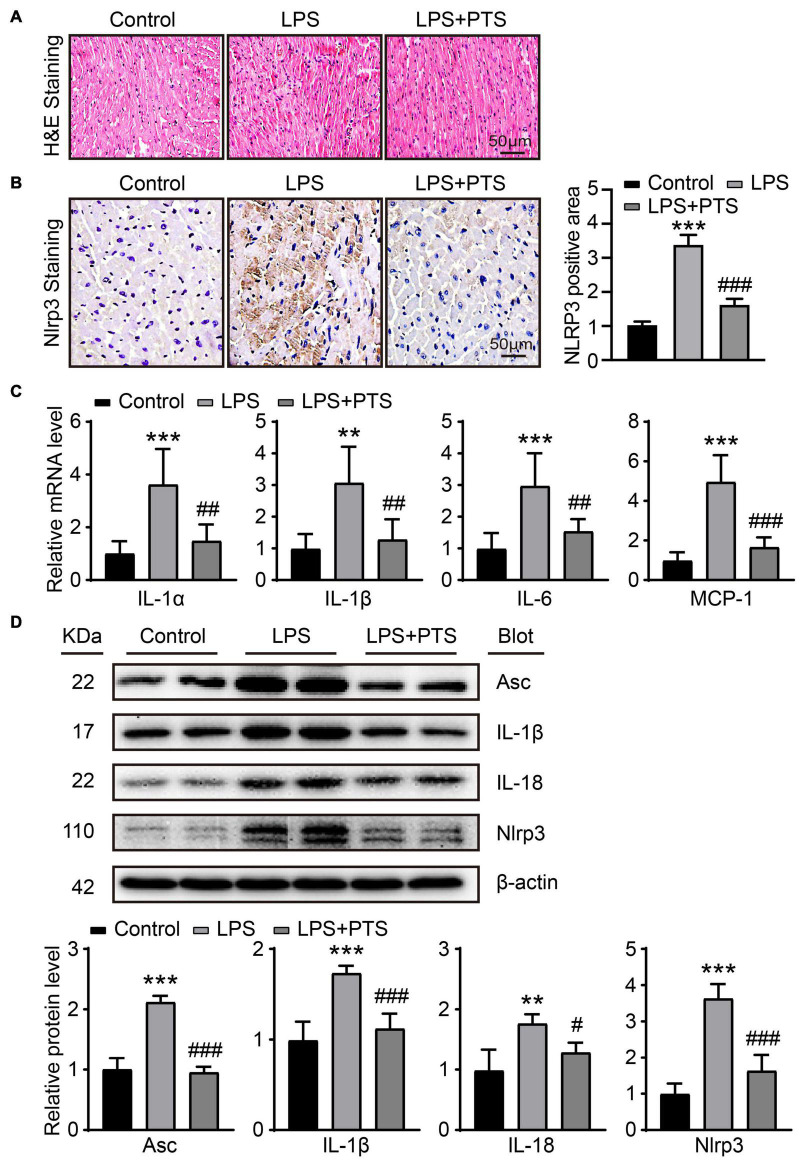
PTS inhibits inflammation in the hearts of mice with LPS-Induced septic mice. **(A)** Representative H&E images of heart tissue from control group, LPS group, and LPS + PTS group. **(B)** Representative NLRP3 of IHC from control group, LPS group, and LPS + PTS group on the left and quantitative data are shown on the right (*n* = 6). **(C)** The mRNA levels of IL-1α, Il-1β, IL-6, and MCP-1 in heart tissue from control group, LPS group, and LPS + PTS group were detected by qPCR (*n* = 6). **(D)** Expressions of ACS, IL-1β, Il-18, and NLRP3 protein in heart tissue were measured by Western blotting analysis (*n* = 4). **P < 0.01, ***P < 0.001 control vs. LPS, ^#^*P* < 0.05, ^##^*P* < 0.01, ^###^*P* < 0.001 LPS vs. LPS + PTS.

Inflammasome signal pathway is an important pathway for LPS to induce myocardial injury. We first verified the inflammatory infiltration by HE staining. The results showed that LPS could promote the production of myocardial tissue inflammation. The addition of PTS could inhibit inflammation (Figure 2A). Next, we used immunohistochemistry to detect the expression of NLRP3 in the heart tissues of each group. The results showed that compared with the control group, the expression of NLRP3 in the LPS group was significantly increased. Compared with the LPS group, the expression of NLRP3 in the LPS + PTS group was significantly decreased (Figure 2B). IL-1α, Il-1β, IL-6, and MCP-1 play an important role in inflammation-related research. In this experiment, we found that compared with the control group, IL-1α, Il-1β, IL-6 and MCP-1 mRNA levels in the LPS group were significantly increased, and compared with the LPS group, the MCP in the LPS + PTS group. The expression of IL-1α, Il-1β, IL-6, and MCP-1 decreased significantly (Figure 2C). Next, we tested the expression of inflammasome-related proteins. Compared with the control group, the levels of ACS, IL-1β, Il-18, and NLRP3 in the LPS group were significantly increased, and compared with the LPS group, the expression of ACS, IL-1β, Il-18, and NLRP3 in the LPS + PTS group decreased significantly (Figure 2D).

### Pterostilbene Inhibits Fibrosis in the Hearts of Mice With Lipopolysaccharide-Induced Septic Mice

**FIGURE 3:**
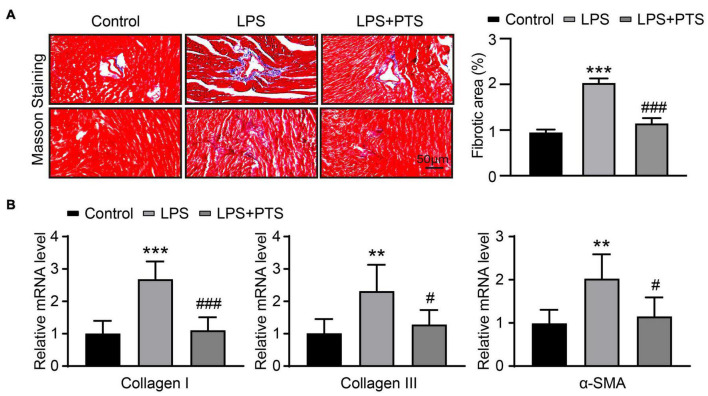
PTS inhibits fibrosis in the hearts of mice with LPS-Induced septic mice. **(A)** Representative of Masson from control group, LPS group, and LPS + PTS group on the left and quantitative data are shown on the right (*n* = 6). **(B)** The mRNA levels of IL-1α, Il-1β, IL-6, and MCP-1 in heart tissue from control group, LPS group, and LPS + PTS group were detected by qPCR (*n* = 6). **P < 0.01, ***P < 0.001 control vs. LPS, ^#^*P* < 0.05, ^###^*P* < 0.001 LPS vs. LPS + PTS.

The Masson staining of mouse heart section is shown in the Figure 3. It showed that the level of fibrosis in the heart of the LPS group was significantly higher than that of the control group, while the level of fibrosis in the LPS + PTS group was significantly lower than the LPS group (Figure 3A). We did the expression of fibrosis-related genes collagen I, collagen III, and α-SMA. Similarly, the expression of collagen I, collagen III, and α-SMA in the LPS group mice were significantly higher than those of the control group, while the collagen I, collagen III, and α-SMA of the LPS + PTS group mice were significantly lower than those of the LPS group (Figure 3B). The results show that PTS could significantly reduce myocardial fibrosis induced by LPS.

### Pterostilbene Reduces Oxidative Stress in the Hearts of Mice With Lipopolysaccharide-Induced Septic Mice

**FIGURE 4:**
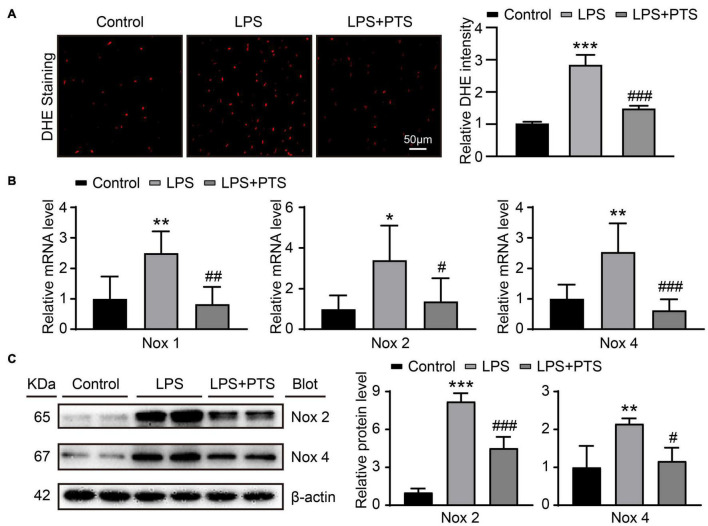
PTS reduces oxidative stress in the hearts of mice with LPS-Induced septic mice. **(A)** Representative of DHE from control group, LPS group, and LPS + PTS group on the left and quantitative data are shown on the right (*n* = 6). **(B)** The mRNA levels of NOX1, NOX2, and NOX4 in heart tissue from control group, LPS group, and LPS + PTS group were detected by qPCR (*n* = 6). **(C)** Expressions of NOX2 and NOX4 protein in heart tissue were measured by Western blotting analysis (*n* = 4). *P < 0.05, **P < 0.01, ***P < 0.001 control vs. LPS, ^#^*P* < 0.05, ^##^*P* < 0.01, ^###^*P* < 0.001 LPS vs. LPS + PTS.

The detection of oxidative stress related indicators in mouse myocardial tissue after LPS injection for 24 h showed that compared with the control group, the expression of DHE in the myocardial tissue of mice in the LPS group was significantly increased, and compared with the LPS group, the expression of DHE in the LPS + PTS group obviously decreased (Figure 4A). Next, we examined the mRNA expression level of oxidative stress-related molecules NOX1, NOX2, and NOX4. The results showed that compared with the control group, the expressions of NOX1, NOX2, and NOX4 in the myocardial tissue of the LPS group were significantly increased, and compared with the LPS group, the expressions of NOX1, NOX2, and NOX4 were significantly reduced in the LPS + PTS group (Figure 4B). Finally, we did the detection of oxidative stress-related molecular protein expression level. The same results showed that compared with the control group, the expression of NOX2 and NOX4 in the myocardial tissue of the LPS group was significantly increased, and compared with the LPS group, the expression of NOX2 and NOX4 was significantly reduced in the LPS + PTS group (Figure 4C).

### Pterostilbene Reduces Oxidative Stress in Lipopolysaccharide-Induced H9C2 Cells

**FIGURE 5:**
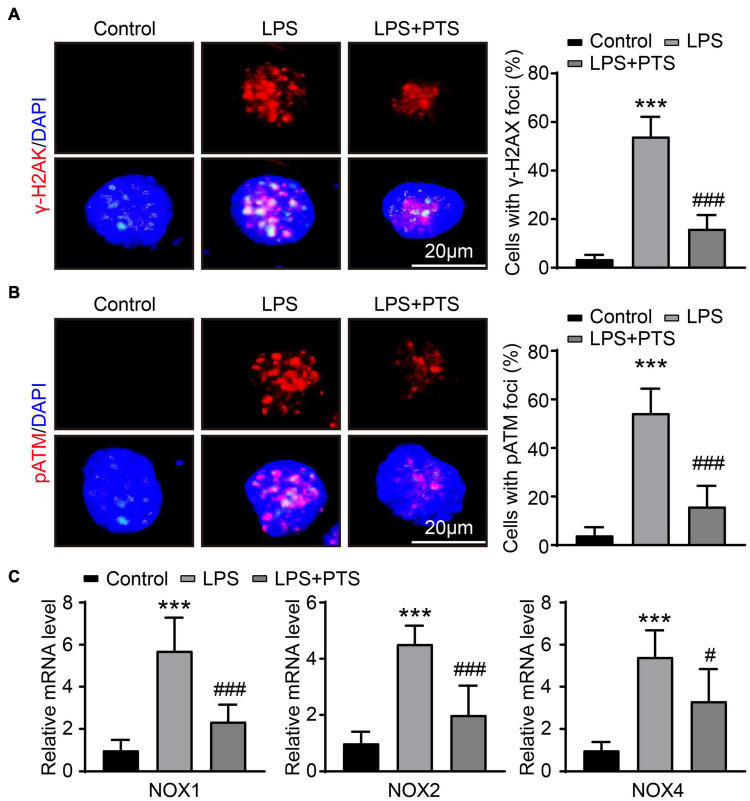
PTS reduces oxidative stress in LPS-Induced H9C2 cells. **(A)** Representative of γ-H2AX and DAPI from control group, LPS group, and LPS + PTS group on the left and quantitative data are shown on the right (*n* = 6). **(B)** Representative of p-ATM and DAPI from control group, LPS group, and LPS + PTS group on the left and quantitative data are shown on the right (*n* = 6). **(C)** The mRNA levels of NOX1, NOX2, and NOX4 in heart tissue from control group, LPS group, and LPS + PTS group were detected by qPCR (*n* = 6). ***P < 0.001 control vs. LPS, ^#^*P* < 0.05, ^###^*P* < 0.001 LPS vs. LPS + PTS.

We had tested that PTS could protect LPS-induced myocardial damage *in vivo*. Next, we tested whether PTS protected H9C2 cells from LPS stimulation *in vitro*. First, we did immunofluorescence detection of γ-H2AX and p-ATM. The results showed that compared with the control group, LPS could significantly increase the expression of γ- H2AX and p-ATM, while compared with the LPS group, LPS + PTS could significantly inhibit the expression of γ-H2AX and p-ATM (Figures 5A,B). Next, we tested the mRNA expression level of oxidative stress-related molecules NOX1, NOX2, and NOX4. The results showed that compared with the control group, the expressions of NOX1, NOX2, and NOX4 in the myocardial tissue of the LPS group were significantly increased, and compared with the LPS group, the expression of NOX1, NOX2, and NOX4 were significantly reduced in the LPS + PTS group (Figure 5C).

### Pterostilbene Inhibits Inflammasome-Related Pathways in Lipopolysaccharide-Induced H9C2 Cells

**FIGURE 6:**
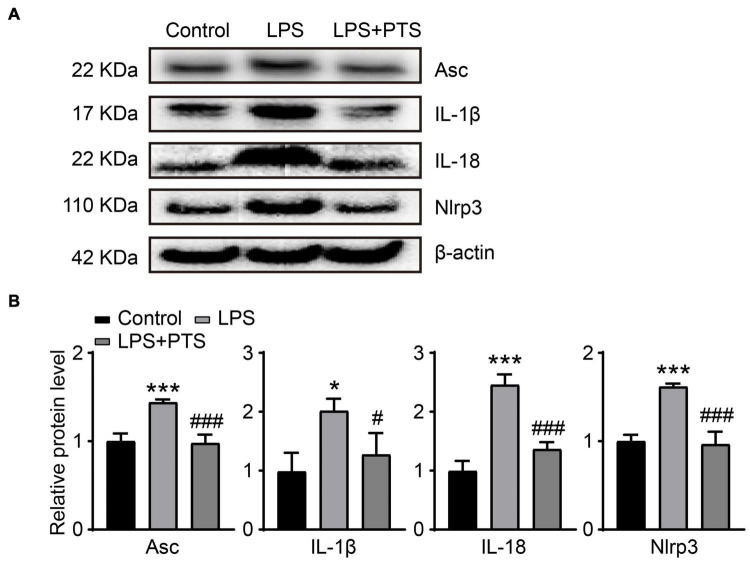
PTS inhibits Inflammasome-related pathways in LPS-Induced H9C2 cells. **(A)** Expressions of ACS, IL-1β, Il-18, NLRP3 and β-actin protein in heart tissue were measured by Western blotting analysis. **(B)** Relative quantitative analysis of ACS, IL-1β, Il-18, and NLRP3 (*n* = 3). *P < 0.05, ***P < 0.001 control vs. LPS, ^#^*P* < 0.05, ^###^*P* < 0.001 LPS vs. LPS + PTS.

Lipopolysaccharide mainly regulates cell death through the pyrolysis and inflammasome pathway. We verified the expression of key proteins in Inflammasome-related pathways *in vitro* experiments. The results show that compared with the control group, LPS could significantly stimulate the expression of ACS, IL-1β, Il-18, and NLRP3, while compared with the LPS group, LPS + PTS could significantly inhibit ACS, IL-1β, Il-18, and NLRP3 expression (Figures 6A,B).

## Discussion

The pathogenesis of sepsis-induced myocardial dysfunction is currently unclear. According to reports, persistent inflammation, increased production of reactive oxygen species, mitochondrial dysfunction, and autonomic nervous system disorders have all been implicated (Lin et al., 2020; Song et al., 2020). Therefore, treatments to reduce myocardial dysfunction caused by sepsis are limited. The treatment of this deadly disease is still based on antibiotics and supportive therapy. Therefore, it is necessary to reveal the mechanism of sepsis induced myocardial injury and explore new therapeutic targets (Poustchi et al., 2021).

Pterostilbene is a natural compound with a structure similar to resveratrol and is a methylated derivative of resveratrol. Pterostilbene has anti-tumor, anti-inflammatory, antioxidant, anti-aging, and cardiovascular protection effects. Relevant animal experiments have shown that Pterostilbene fat solubility is higher, the bioavailability is nearly four times that of resveratrol, and it has a good prevention and treatment effect on metabolic diseases and acute and chronic cardiovascular diseases, and is one of the hot compounds currently studied (Ma et al., 2019). It has shown that LPS could lead to the activation of the innate immune system and at the same time activate the inflammatory pathway of cardiomyocytes (Rathinam et al., 2019). The oxidative stress response produced by it could damage the cells and lead to myocardial damage. When the dose of LPS injected into the animal is large enough, the animal will show physiological and biochemical changes. After the use of drugs to inhibit the related myocardial inflammatory pathway, the mouse heart dysfunction caused by LPS could also be improved to a certain extent (Abd et al., 2021; Dai et al., 2021a).

Inflammatory bodies play a key role in the release of pro-inflammatory cytokines and chemokines such as IL-1β and IL-18. NLRP3 is a member of the NOD-like receptor (NOD-like receptor, NLR) family. It forms an inflammasome that forms a variety of protein complexes with ASC and pro-caspase-1 (Dai et al., 2021b). The activation of NLRP3 inflammatory bodies could activate caspase-1. The activated caspase-1 cleaves the pre-IL-1β and pre-IL-18 production, and releases IL-1β and IL-18, thereby inducing an inflammatory response (Guo et al., 2020; Qiu et al., 2021). In this article, we found that LPS could promote the expression of inflammasome-related proteins and genes. It is proved that after LPS treatment, pyroptosis and inflammasome occurred in the myocardial tissue of mice. After PTS treatment, the expression of ASC, NLRP3 and IL-1β and other marker factors were significantly downregulated. This shows that PTS could inhibit the production of inflammasomes induced by LPS.

After myocardial cell injury, it will recruit the production of inflammatory factors, and then fibrosis will occur. The concentration of collagen in the heart tissue is significantly increased (Yin et al., 2012; Han et al., 2017). PTS has been shown to inhibit or reduce the production of fibrosis in myocardial ischemia and reperfusion, liver injury and lung injury (Lv et al., 2015; Couto et al., 2018; Kosuru et al., 2018; Lacerda et al., 2020; Zhang et al., 2021). In this article, we found that the fibrosis level in the myocardial tissue after PTS treatment has been effectively controlled by the fibrosis markers collagen I and collagen III and Masson.

Oxidative stress plays an important role in organ damage caused by sepsis. Excessive production of active substances and/or free radicals is the destruction of oxygen utilization by cells and the limitation of oxygen delivery by tissues (Sies et al., 2017). NADPH oxidase reduced nicotinamide adenine dinucleotide phosphate oxidase (NOX) is widely expressed in the cardiovascular system, and is different from other enzymes that produce reactive oxygen species in enzymatic reactions. NOX is an enzyme that produces reactive oxygen species. In the pathological state of myocardial remodeling, NOX imbalance is one of the important causes of cardiac oxidative stress, and long-term oxidative stress could lead to a series of cardiac changes such as myocardial hypertrophy and myocardial fibrosis (Chen et al., 2020). NOX2 mainly catalyzes the production of O_2_-(Do et al., 2021), while NOX4 mainly produces a large amount of H_2_O_2_ (Steinhorn et al., 2017). The main function of NOX1 is to produce superoxide and quickly convert it into hydrogen peroxide. NOX1 could be activated by a variety of agonists such as pro-inflammatory factors (TNF-α, IL-1β), oxidized low-density lipoprotein, etc. NOX4 is the most expressed NOX family member in the cardiovascular system. Under pathological conditions such as heart failure and cardiac pressure overload, NOX4 in the mitochondria of cardiomyocytes is upregulated, reactive oxygen species increase, mitochondrial proteins are oxidized, and electrons caused by mitochondrial dysfunction. Leakage leads to an increase in reactive oxygen species, so as an important source of mitochondrial oxidative stress, NOX4 is a key mediator of oxidative stress and cardiac dysfunction during cardiac pressure overload (Cao et al., 2019). In this article, we have proved through *in vivo* and *in vitro* experiments that PTS could inhibit the expression of NOXs induced by LPS.

There are still some limitations of this article at this stage. Although we found that Pterostilbene inhibits LPS-induced myocardial injury through the oxidative stress pathway and the inflammasome pathway. However, we only discovered this phenomenon by detecting the final point of the pathway, and did not specifically study which proteins regulate these two mechanisms. Next, it may be necessary to further define the role of PTS in LPS-induced myocardial injury by adding different oxidative stress inhibitors or inflammasome inhibitors. Identify which gene or protein plays an important role by proteomics or PCR assay.

**FIGURE 7:**
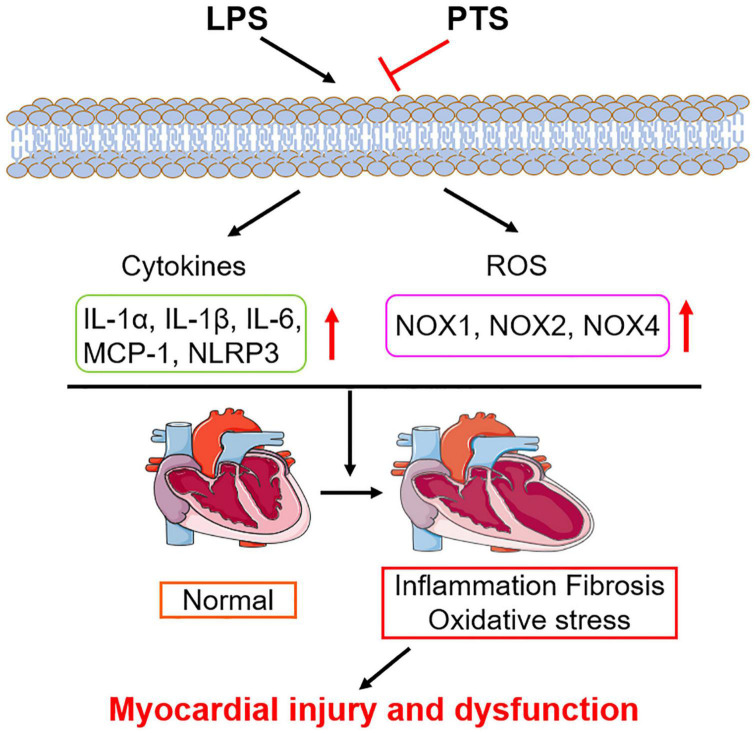
Schematic diagram of the mechanism: pterostilbene inhibits LPS-induced myocardial injury.

All in all, this study clearly shows that PTS could protect the heart function by downregulating the production of inflammation and oxidative stress (Figure 7). These findings indicate that PTS may be a beneficial treatment strategy for cardiac dysfunction caused by sepsis and could be used in the treatment of intensive care units.

## Data Availability Statement

The raw data supporting the conclusions of this article will be made available by the authors, without undue reservation.

## Ethics Statement

The animal study was reviewed and approved by the Dalian Medical University.

## Author Contributions

LZ, XJ, and JCY investigated, acquired, analyzed, interpreted, and visualized the data, conceived and designed the study, and drafted the manuscript. JY and JNY revised the manuscript, acquired funding, and supervised the study. All authors contributed to the article and approved the submitted version. The authors declare that all data were generated in-house and that no manuscript mill was used.

## Conflict of Interest

The authors declare that the research was conducted in the absence of any commercial or financial relationships that could be construed as a potential conflict of interest.

## Publisher’s Note

All claims expressed in this article are solely those of the authors and do not necessarily represent those of their affiliated organizations, or those of the publisher, the editors and the reviewers. Any product that may be evaluated in this article, or claim that may be made by its manufacturer, is not guaranteed or endorsed by the publisher.
